# Inhibition of miR-140-5p attenuates OGD/R-induced neuronal injury by targeting IL1RAPL1

**DOI:** 10.3389/fnmol.2026.1972044

**Published:** 2026-10-23

**Authors:** Yuan-yue Song, Zhu-jun Chen, Heng-yu Yan, Li-li Zeng

**Affiliations:** 1 Department of Neurology and Institute of Neurology, Ruijin Hospital, Shanghai Jiao Tong University School of Medicine, Shanghai, China; 2 Department of Geriatric Medicine, Huashan Hospital, Shanghai Medical College, Fudan University, Shanghai, China

**Keywords:** IL1RAPL1, miR-140-5p, neuroinflammation, neuron, oxygen-glucose deprivation

## Abstract

**Objective:**

Cerebral ischemia–reperfusion (I/R) injury is a major contributor to neuronal damage and long-term neurological dysfunction after stroke. Our previous studies demonstrated that miR-140-5p expression is markedly upregulated following cerebral ischemia and that in vivo overexpression of miR-140-5p exacerbates cognitive impairment, suppresses neurogenesis, and promotes neuronal death in ischemic mouse models; however, its molecular mechanisms remain unclear. This study aimed to investigate the downstream molecular targets of miR-140-5p and to elucidate the mechanisms by which miR-140-5p regulates neuronal injury after I/R.

**Methods:**

We established an oxygen–glucose deprivation/reoxygenation (OGD/R) model in primary cortical neurons and assessed viability via Cell Counting Kit-8 (CCK-8). miR-140-5p expression after OGD/R was examined, and loss-of-function experiments evaluated the effects of miR-140-5p inhibition. Synaptic integrity was assessed by immunofluorescence, and inflammatory cytokines by enzyme-linked immunosorbent assay (ELISA). Proteomic analysis identified potential downstream targets, which were validated via Western blotting and dual-luciferase reporter assays.

**Results:**

We found that miR-140-5p expression was significantly upregulated in mouse cortical neurons after OGD/R. Functional inhibition of miR-140-5p markedly enhanced neuronal survival, promoted synaptic remodeling, and attenuated inflammatory responses following OGD/R injury. Mechanistically, miR-140-5p was shown to directly target *Il1rapl1* and modulate downstream c-Jun N-terminal kinase (JNK) signaling, thereby regulating neuronal viability, inflammation, and synaptic plasticity under ischemia–reperfusion conditions.

**Conclusion:**

This study reveals a novel miR-140-5p–mediated regulatory mechanism in neuronal injury following cerebral ischemia–reperfusion and suggests a potential therapeutic target for ischemic brain injury.

## 1 Introduction

Cerebral ischemia–reperfusion injury (CIRI) is a critical pathological process underlying acute neuronal damage following ischemic stroke and represents a common mechanistic basis for the development and progression of chronic cerebrovascular diseases, post-stroke depression, and vascular dementia (Fang et al., 2019; Sun et al., 2023; Tuo et al., 2021). CIRI refers to the paradoxical exacerbation of tissue injury that occurs after the restoration of blood flow to ischemic brain regions, and it constitutes a major determinant of neurological dysfunction and poor clinical outcomes after ischemic stroke (Hansen et al., 2025). The pathophysiological mechanisms of CIRI are complex and multifactorial, involving excessive generation of reactive oxygen species, intracellular calcium overload, inflammatory responses, and subsequent neuronal apoptosis (Jurcau and Ardelean, 2022; Lochhead et al., 2024). Despite extensive experimental and clinical investigations, effective therapeutic strategies specifically targeting ischemia–reperfusion–induced brain injury remain limited in current clinical practice.

MicroRNAs (miRNAs), a class of short (approximately 18–25 nucleotides) non-coding single-stranded RNA molecules, have emerged as critical post-transcriptional regulators of gene expression networks and promising biomarkers for early diagnosis, prognosis evaluation, and treatment monitoring in cerebral I/R injury and related neurological diseases (Gendosz de Carrillo et al., 2025; Zhao et al., 2025). Our previous clinical study identified a significant upregulation of miR-140-5p in the plasma of patients with post-stroke depression (Liang et al., 2019). This upregulation was further associated with pathogenic functions in vivo: hippocampal overexpression of miR-140-5p exacerbated cognitive impairment, suppressed neurogenesis, and promoted neuronal death and synaptic protein loss in a mouse model of bilateral common carotid artery occlusion (BCCAO) (Liang et al., 2022). While these findings suggest a critical role of miR-140-5p in stroke-related pathology, its cell-autonomous functions and precise molecular mechanisms within neurons remain unclear. Therefore, the present study aims to elucidate the specific role and regulatory mechanisms of miR-140-5p in neurons using in vitro models, in order to explore its potential as a novel therapeutic target for ischemic brain injury.

Specifically, we sought to characterize the neuronal effects of miR-140-5p and identify the molecular mechanisms underlying its role in ischemia–reperfusion-induced neuronal injury. We employed an unbiased proteomic approach to identify its downstream targets in primary neurons subjected to oxygen-glucose deprivation/reoxygenation (OGD/R). Candidate targets and associated signaling pathways were subsequently validated through a combination of biochemical and molecular techniques, including Western blotting, immunofluorescence, and dual-luciferase reporter assays. We hypothesized that miR-140-5p exacerbates neuronal I/R injury by directly suppressing key neuroprotective or synaptic proteins, thereby activating a detrimental signaling cascade. The elucidation of this axis is expected to not only advance our understanding of post-ischemic neuronal death but also provide a novel conceptual framework for targeted therapeutic intervention.

## 2 Materials and methods

### 2.1 Cell culture

The primary culture of neurons in the cerebral cortex was obtained from the embryonic brains of E16-18 pregnant C57BL/6 mice (Leigen, Shanghai, China). Under a microscope, embryonic brains were carefully dissected, removing brain membranes and blood vessels. The brain tissues were then digested with 0.125% Trypsin-EDTA at 37 °C for 6 min, with fetal bovine serum (FBS) used to halt digestion. The resulting cell suspension was filtered using a 40 μm cell sieve and seeded into culture dishes at a density of 4 × 10^5^ cells/mL. Neurons were maintained in serum-free conditions using the B27 supplement, supplemented with 1% Glutamax(Gibco, CA) and 1% Penicillin/Streptomycin(P/S) (Gibco, CA).

### 2.2 Oxygen-Glucose Deprivation/reoxygenation (OGD/R) model

Experiments were performed only when cells were in good condition (viability > 90%, no contamination). The OGD/R model was employed to simulate cerebral ischemia-reperfusion in vitro. Firstly, the OGD chamber was wiped with 75% ethanol and sterilized under ultraviolet light for 30 min. Then, the cell culture medium was replaced with glucose-free dulbecco’s modified Eagle medium (DMEM) (Gibco, CA). Subsequently, the culture plate was placed into the OGD chamber along with an oxygen concentration meter, and a mixture of 95% N2 and 5% CO2 was introduced into the chamber. The process continued until the oxygen concentration dropped to less than 0.5%, and the chamber was sealed for several hours. Primary cortical neurons were then returned to normal oxygen and glucose conditions. Finally, we selected 8 h of OGD followed by 48 h of reoxygenation as the experimental condition.

### 2.3 Cell viability assay

Cell viability was assessed using a Cell Counting Kit-8 (CCK-8) assay. Briefly, following the indicated treatments, the culture medium was replaced with fresh medium containing 10% (v/v) CCK-8 reagent. The cells were then incubated at 37 °C for 2 h, and the absorbance was measured at 450 nm using a microplate reader. Cell viability was expressed relative to that of the corresponding control group.

### 2.4 Construction of miR-140-5p lentiviral expression vector and cell transfection

Lentiviral vectors were used to modulate miR-140-5p expression. For miR-140-5p overexpression, a lentiviral construct expressing the primary miR-140 transcript (pri-miR-140) under the control of a cytomegalovirus (CMV) promoter was used (LV-miR-140-5p). For miR-140-5p inhibition, a lentiviral vector expressing a tough decoy (TuD) sequence specifically targeting mature miR-140-5p driven by the U6 promoter was employed (LV-anti-miR-140-5p). A scrambled sequence lacking complementarity to known mammalian miRNAs was used as a negative control (LV-scramble). All lentiviral vectors were packaged and provided by OBio Technology (Shanghai, China) and stored at −80 °C until use. Primary cortical neurons were transduced with lentiviral particles at a multiplicity of infection (MOI) of 10. Transduction efficiency was assessed 72 h post-transduction by green fluorescent protein (GFP) fluorescence and further confirmed by quantitative real-time PCR (qRT-PCR) analysis of miR-140-5p expression. Stable HT22 cell lines with sustained miR-140-5p overexpression or inhibition were generated using the corresponding lentiviral constructs. Cells were transduced at an MOI of 40, followed by puromycin selection (2 µg/mL) initiated 48 h after transduction. After 10–14 days, puromycin-resistant cell populations were expanded and maintained in medium containing 1 µg/mL puromycin. Stable modulation of miR-140-5p expression was verified by qRT-PCR.

### 2.5 Reverse transcription and quantitative real-time PCR (qRT-PCR)

RNA extraction was performed using the Trizol(Invitrogen, CA) method, and RNA concentration was measured with a NanoDrop1000 Spectrophotometer(Thermofisher, CA). Subsequently, 500 ng of RNA samples from each group were used for reverse transcription with the miRNA First Strand cDNA Synthesis kit(Vazyme, Nanjing, China). Polymerase chain reaction (PCR) amplification was conducted using the miRNA Universal SYBR qPCR Master Mix kit (Vazyme, Nanjing, China) on a real-time RT-PCR system(7900 HT, ABI, CA, USA). The relative expression of miR-140-5p was calculated using the 2 raised to the power of −∆∆CT formula compared with the U6 internal reference gene (Table 1).

**Table 1:** Primer sequences for qRT-PCR.

Gene of interest	Sequences
miRNA-140-5p-F	CGCGCAGTGGTTTTACCCTA
miRNA-140-5P-R	AGTGCAGGGTCCGAGGTATT
miRNA-140-5P-RT	GTCGTATCCAGTGCAGGGTCCGAGGTATTCGCACTGGATACGACCTACCA
U6-f	CTCGCTTCGGCAGCACA
U6-R	AACGCTTCACGAATTTGCGT
U6-RT	AACGCTTCACGAATTTGCGT

F, forward; R, reverse; RT, reverse transcription primer.

### 2.6 Western blot analysis

Total protein was extracted from neurons using radioimmunoprecipitation assay (RIPA) lysis buffer (Beyotime) containing 1% protease and phosphatase inhibitor cocktails (both from Epienzyme). Protein concentrations were determined with a bicinchoninic acid (BCA) assay kit (Beyotime). Equal amounts of protein (20–30 µg per lane) were separated by sodium dodecyl sulfate–polyacrylamide gel electrophoresis (SDS-PAGE) and transferred onto 0.2 µm polyvinylidene fluoride (PVDF) membranes. After blocking with 10% bovine serum albumin (BSA), membranes were incubated overnight at 4 °C with the following primary antibodies: β-actin (1:1000, proteintech), p-JNK (1:1000, CST), JNK (1:1000, CST), *Il1rapl1* (1:1000, proteintech), PSD95 (1:1000; Millipore), and p-PSD95 (phospho-S295) (1:1000, abcam). Membranes were then probed with horseradish peroxidase (HRP)-conjugated goat anti-rabbit (1:10000, proteintech) or goat anti-mouse (1:5000, proteintech) secondary antibodies for 1 h. Protein bands were visualized using an enhanced chemiluminescence (ECL) substrate (Beyotime) and imaged with a Chemiluminescence Imager (Bio-Rad). Band intensities were quantified with ImageJ software (National Institutes of Health) and normalized to β-actin.

### 2.7 Immunofluorescence and terminal deoxynucleotidyl transferase dUTP nick-end labeling (TUNEL) assay

Cells cultured on sterile cover slips were fixed with 4% paraformaldehyde for 10 min and permeabilized with 0.3% Triton X-100 for 10 min. After blocking with 3% BSA for 1 h at room temperature, the following primary antibodies were incubated overnight at 4 °C: mouse Neuron(1:200, proteintech, China), rabbit PSD-95(1:200, Servicebio, Wuhan, China), mouse Synaptophysin(1:200, proteintech, China). Subsequently, the secondary antibody, Alexa 488/555-conjugated donkey anti-rabbit/mouse(1:400, Beyotime), was applied. For TUNEL assay, after fixation, TUNEL reagent (Beyotime, China) was applied to the slides, then incubated at 37 °C for 1 h.Finally, 12 µL of mounting medium containing 4′,6-diamidino-2-phenylindole (DAPI) was added to observe fluorescence under a laser scanning confocal microscope. After each addition of new reagent, the cells were washed with PBS three times for 5 min.

### 2.8 Enzyme-linked immunosorbent assay (ELISA)

The concentrations of inflammatory cytokines in cell culture supernatants were measured using commercial ELISA kits (Yeasen, China). Briefly, supernatants were collected by centrifugation to remove cellular debris. Following the manufacturer’s protocol, samples and standards were incubated in pre-coated plates. After development, the absorbance was measured, and sample concentrations were calculated from a standard curve.

### 2.9 Proteomic analysis and candidate protein selection

Proteomic analysis was performed using samples from the indicated experimental groups. To identify proteins potentially associated with miR-140-5p inhibition under OGD/R conditions, differentially expressed proteins between the inhibitor-OGD/R and inhibitor-NC-OGD/R groups were screened using the criteria of log2(fold change [FC]) > 1 and *P* < 0.05. The differentially expressed proteins were subsequently subjected to Kyoto Encyclopedia of Genes and Genomes (KEGG) pathway enrichment analysis, with particular attention to pathways associated with neuronal survival, apoptosis, and synaptic function. In parallel, potential target genes of miR-140-5p were predicted using TargetScan. The proteomic results were then integrated with the TargetScan prediction to identify candidate downstream targets of miR-140-5p. Among the candidates, IL1RAPL1 was selected for further investigation based on its differential expression pattern, its association with synaptic function, and its predicted miR-140-5p binding site. The direct interaction between miR-140-5p and *Il1rapl1* was subsequently validated using a dual-luciferase reporter assay.

### 2.10 Dual-luciferase reporter assay

MiRNAs regulate gene expression mainly through binding to complementary sequences in the 3′ untranslated region (3′UTR) of target mRNAs. To validate the interaction between miR-140-5p and *Il1rapl1*, the full-length 3′UTR of *Il1rapl1* was cloned into the pGL4 firefly luciferase reporter vector (ObiO Co., Ltd., Shanghai, China). Mutant constructs containing disrupted miR-140-5p binding sites were generated by site-directed mutagenesis. HEK-293T cells were used for luciferase reporter assays and co-transfected with 200 ng of firefly luciferase reporter plasmid, 10 ng of Renilla luciferase control plasmid, and 100 nmol/L synthetic miR-140-5p mimic or negative control using Lipofectamine 2000. Luciferase activity was measured using a dual-luciferase reporter assay system, and relative luciferase activity was calculated as the ratio of Renilla luciferase to firefly activity.

### 2.11 Statistical analysis

Results are from at least three independent biological replicates (*n* ≥ 3) and are presented as mean ± SD. For comparisons involving more than two groups, one-way analysis of variance (ANOVA) with Student–Newman–Keuls post hoc analysis was applied; comparisons between two groups were assessed using Student’s t-test. Statistical significance was defined as *P* < 0.05. GraphPad Prism 7 (GraphPad Software, CA, USA) was used for all analyses.

## 3 Results

### 3.1 OGD/R model in primary neurons, time-dependent miR-140-5p upregulation, and lentiviral knockdown system

To establish an appropriate OGD/R model, primary neurons were exposed to OGD for different durations (4, 8, and 12 h; Figure 1A) and cell viability was assessed. Neuronal survival decreased in a time-dependent manner, reaching approximately 50% after 8 h of OGD; therefore, 8 h was selected for subsequent experiments. miR-140-5p expression was further evaluated at different reoxygenation time points (0, 12, 24, and 48 h; Figure 1B) and was significantly increased after 48 h of reoxygenation, which was chosen as the optimal reperfusion duration. Lentivirus-mediated knockdown and overexpression of miR-140-5p were successfully established in primary mouse cortical neurons and HT22 cells, as confirmed by qRT-PCR (Figures 1C–E). In the OGD/R-induced neuronal injury model, miR-140-5p expression was significantly upregulated, and its levels were effectively modulated by LV-miR-140-5p or LV-anti-miR-140-5p infection (Figure 1F).

**Figure 1:**
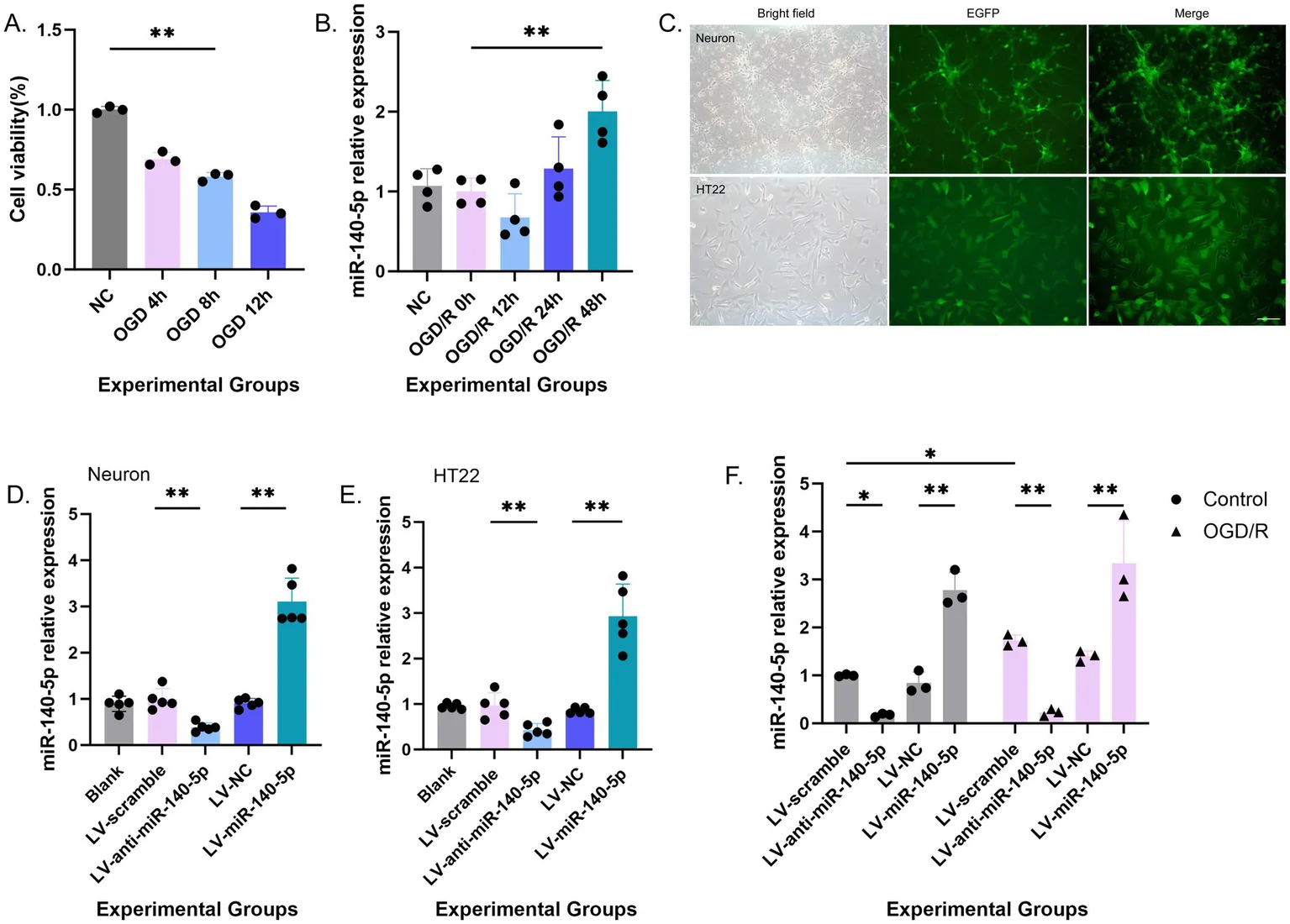
miR-140-5p expression is upregulated in neurons after OGD/R. **(A)** Cell viability of primary mouse cortical neurons after oxygen–glucose deprivation (OGD), as assessed by the CCK-8 assay. **(B)** Relative expression levels of miR-140-5p in primary cortical neurons at different reoxygenation time points after OGD, determined by qRT-PCR. **(C)** Representative immunofluorescence images showing lentiviral transduction efficiency in primary cortical neurons and HT22 cells (GFP). Scale bar = 50 μm. **(D,E)** qRT-PCR analysis of miR-140-5p expression in primary cortical neurons **(D)** and HT22 cells **(E)** after lentiviral-mediated overexpression or inhibition of miR-140-5p. **(F)** qRT-PCR analysis of miR-140-5p expression in primary cortical neurons infected with lentivirus after OGD treatment.

### 3.2 Inhibition of miR-140-5p attenuates apoptosis, enhances synaptic integrity, and suppresses inflammation after OGD/R

Cell viability was assessed using the CCK-8 method. The results indicated that the viability of primary cortical neurons and HT22 cells induced by OGD/R was significantly increased in the LV-anti-miR-140-5p group (*p* < 0.05) (Figures 2A,B). TUNEL staining can be used to directly assess the destructive capacity of apoptosis on nuclear DNA, thereby indirectly indicating the phenomenon of apoptosis. The results showed a significant increase in TUNEL-positive cells in the LV-scramble-OGD/R group, while inhibition of miR-140-5p significantly reduced the number of TUNEL-positive cells (*P* < 0.05), suggesting a decrease in the rate of apoptosis (Figures 2C,D). We performed immunofluorescence staining on primary cortical neurons from mice to observe synaptic changes. The results showed that compared to the LV-scramble-OGD/R group, the relative fluorescence intensity of PSD-95 (a postsynaptic marker) and Synaptophysin (a presynaptic marker) significantly increased in the LV-anti-miR-140-5p-OGD/R group (Figures 2E–G). This indicates that inhibiting miR-140-5p levels after OGD/R can enhance synaptic plasticity, upregulate Synaptophysin expression, promote synaptic structural repair, and potentially improve abnormal dendritic branching and dendritic spine density. WB results showed that after inhibiting miR-140-5P, both PSD95 and p-PSD95 protein levels increased, suggesting that inhibiting miR-140-5P may protect synaptic function by upregulating the expression of PSD95 and p-PSD95, thereby alleviating OGD/R-induced neuronal damage (Figures 2H–J). We also found that inhibition of miR-140-5p reduced IL-6 levels and increased IL-10 expression following OGD/R (Figures 2K,L).

**Figure 2:**
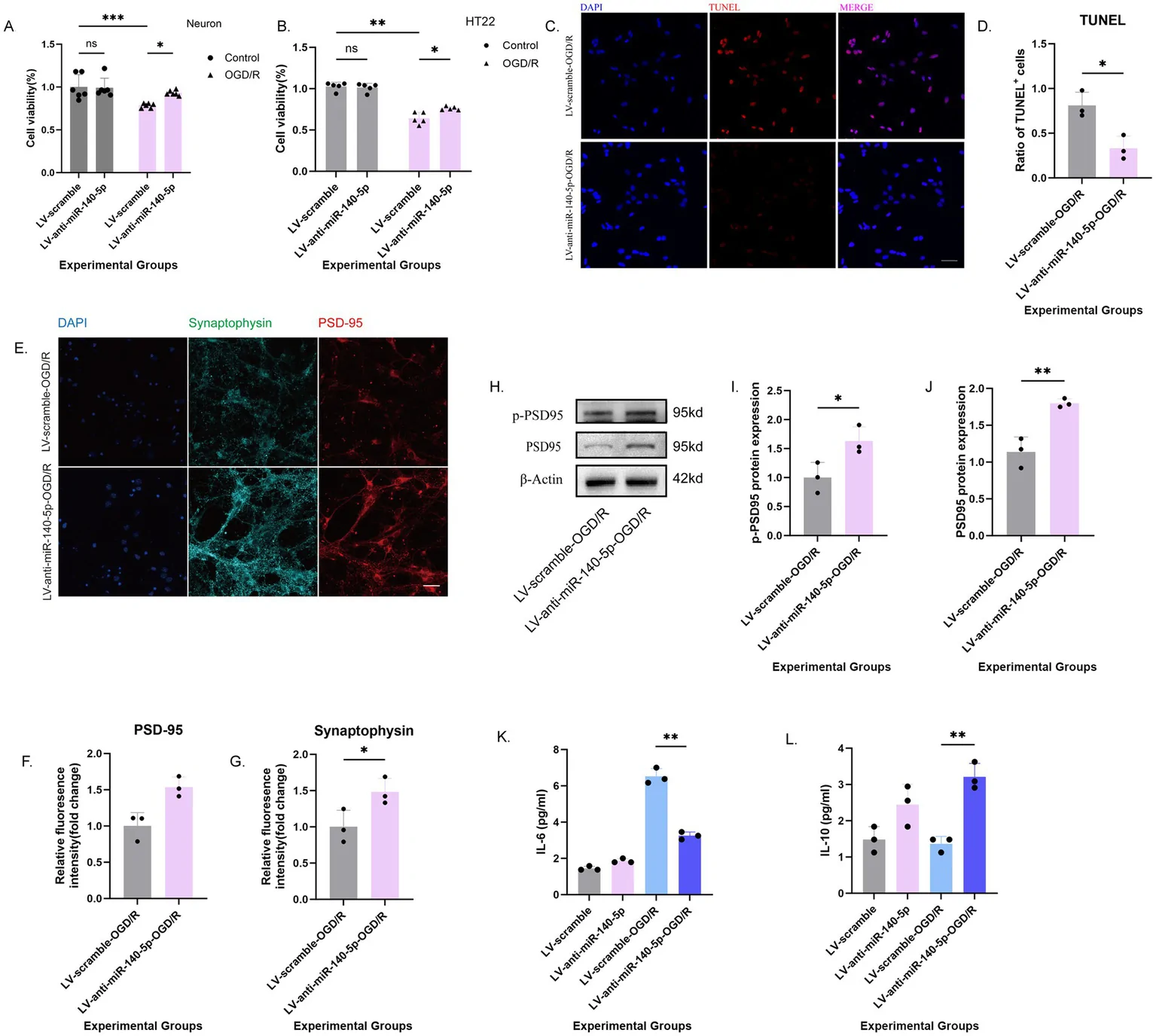
Inhibition of miR-140-5p alleviates OGD-induced neuronal injury and synaptic impairment. **(A,B)** Cell viability of lentivirus-transduced primary cortical neurons **(A)** and HT22 cells **(B)** following OGD, as measured by the CCK-8 assay. **(C,D)** Representative TUNEL staining images of primary cortical neurons after OGD and quantitative analysis of TUNEL-positive cells. **(E–G)** Representative immunofluorescence staining of primary cortical neurons following lentiviral infection, showing changes in neuronal and synaptic markers. **(H–J)** Western blot analysis of p-PSD95 and total PSD95 protein expression in primary cortical neurons after OGD. **(K)** ELISA measurement of IL-6 levels in cell culture supernatants. **(L)** ELISA measurement of IL-10 levels in cell culture supernatants.

### 3.3 Integrated proteomics and bioinformatics analysis reveals downstream targets of miR-140-5p

To explore synapse-related targets of miR-140-5p under OGD/R conditions, proteomic data were visualized using a heatmap to identify differentially expressed synaptic proteins between the LV-anti-miR-140-5p–OGD/R group and the LV-scramble–OGD/R group. These proteins were intersected with predicted miR-140-5p targets from TargetScan, and IL1RAPL1 was selected for further analysis (Figure 3A). KEGG pathway enrichment analysis revealed that the differentially expressed proteins were significantly enriched in several pathways, with apoptosis identified as a significantly enriched pathway (Figure 3B). TargetScan analysis predicted a conserved binding site for miR-140-5p within the 3′UTR of *Il1rapl1* (Figure 3C). To validate this interaction, dual-luciferase reporter constructs containing either the wild-type or mutant *Il1rapl1* 3′UTR were generated and co-transfected with miR-140-5p mimic or negative control into HEK293T cells. The miR‑140‑5p mimic significantly reduced luciferase activity of the wild‑type *Il1rapl1* 3′UTR (Figure 3D), suggesting *Il1rapl1* is a target of miR‑140‑5p (Figure 3D). Western blot analysis showed that inhibition of miR-140-5p significantly increased Il1rapl1 protein expression in mouse cortical neurons compared with controls (Figures 3E, F).

**Figure 3:**
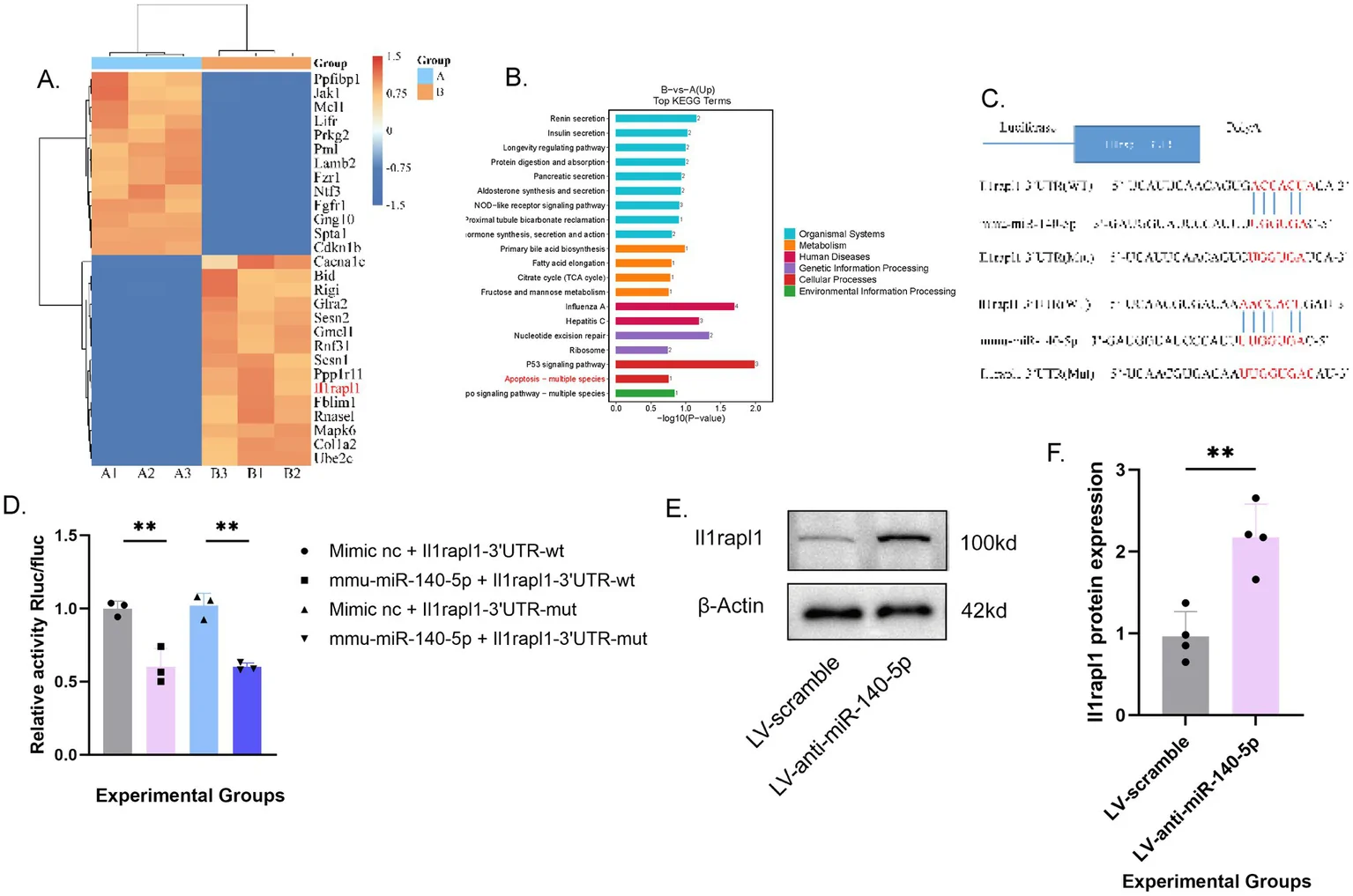
IL1RAPL1 is a direct target of miR-140-5p. **(A)** Heatmap showing differentially expressed synapse-related proteins between LV-scramble-OGD/R (group A) and LV-anti-miR-140-5p-OGD/R (group B). **(B)** KEGG pathway enrichment analysis of differentially expressed genes. **(C)** Predicted binding sites between miR-140-5p and the 3′-UTR of *Il1rapl1* mRNA identified using the TargetScan database. **(D)** Dual-luciferase reporter assay validating the direct interaction between miR-140-5p and the *Il1rapl1* 3′-UTR. **(E,F)** Western blot analysis of IL1RAPL1 protein expression levels in cells following miR-140-5p modulation.

### 3.4 miR-140-5p regulates OGD/R-induced JNK signaling and apoptosis in mouse neuronal cells

Western blot analysis showed that, compared with the LV-scramble-OGD/R group, inhibition of miR-140-5p significantly decreased Bax protein levels and the cleaved caspase-3/caspase-3 ratio, while significantly increasing Bcl-2 expression in HT22 cells (*p* < 0.05) (Figures 4A–D). Furthermore, OGD/R treatment significantly increased the p-JNK/JNK ratio compared with the control group (Figures 4E,F). In contrast, inhibition of miR-140-5p significantly reduced the p-JNK/JNK ratio under OGD/R conditions (*p* < 0.05) (Figures 4G,H). To further confirm that miR-140-5p exerts its effects through the JNK pathway, we performed a rescue experiment using the JNK-specific inhibitor SP600125 in miR-140-5p-overexpressing neurons subjected to OGD/R. We observed that overexpression of miR-140-5p exacerbated OGD/R-induced neuronal apoptosis and JNK activation, whereas these effects were significantly attenuated by co-treatment with the JNK inhibitor SP600125 (Figures 4I,J).

**Figure 4:**
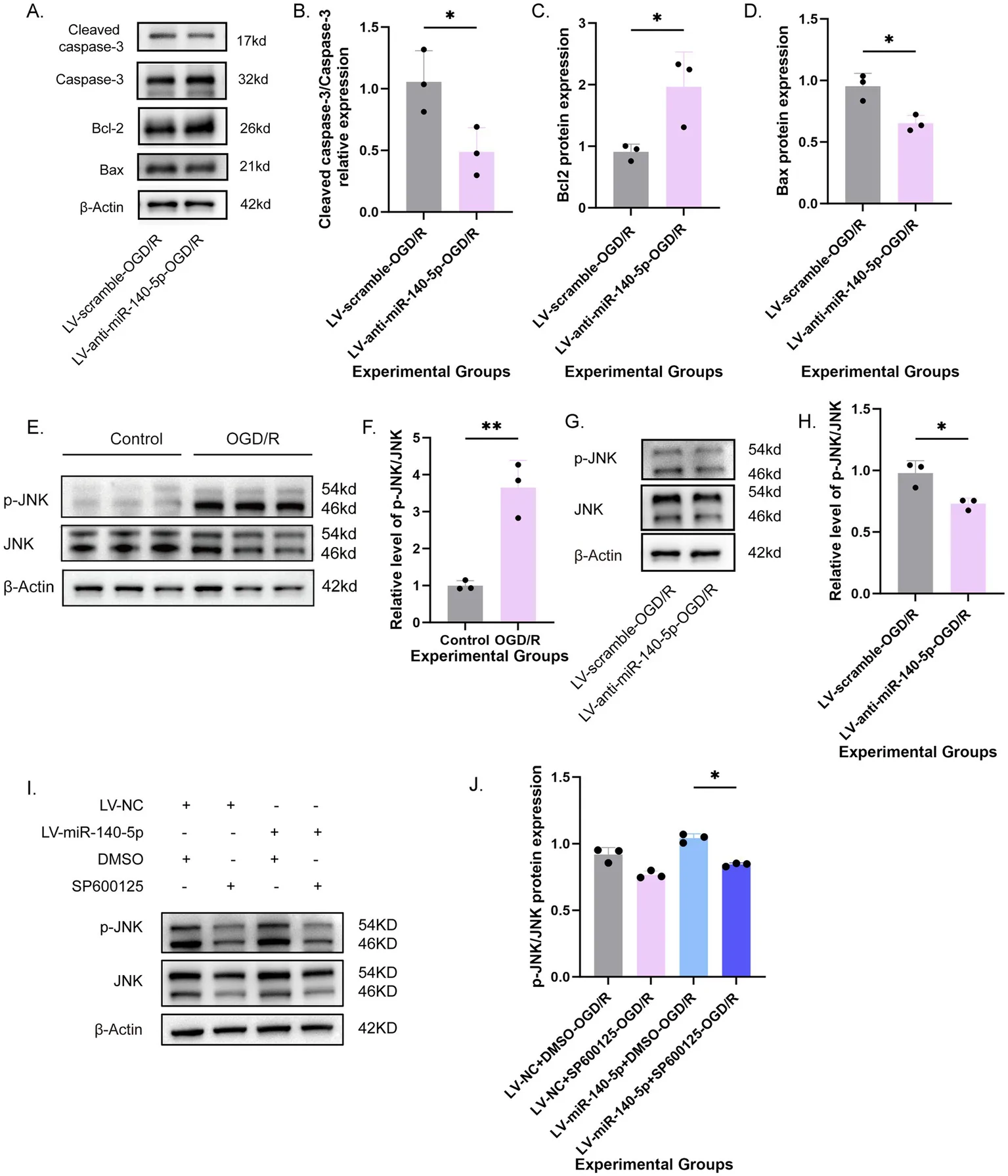
miR-140-5p regulates neuronal apoptosis through the JNK signaling pathway. **(A–D)** Western blot analysis of apoptosis-related proteins in HT22 cells after OGD/R treatment. **(E,F)** Western blot analysis of phosphorylated JNK (p-JNK) and total JNK protein expression. **(G,H)** Western blot analysis of p-JNK/JNK expression in HT22 cells after OGD/R following miR-140-5p inhibition. **(I,J)** Effects of the JNK pathway inhibitor SP600125 on JNK signaling, as determined by Western blot analysis.

## 4 Discussion

In this study, we demonstrated for the first time that miR-140-5p directly targets IL1RAPL1 and regulates inflammation, neuronal death, and synaptic plasticity in OGD/R-induced neuronal injury through activation of the JNK signaling pathway. These findings identify a novel miR-140-5p–IL1RAPL1–JNK regulatory axis and provide new mechanistic insights into ischemia–reperfusion (I/R)-induced neuronal dysfunction.

I/R injury is a key pathological process following ischemic stroke and is increasingly recognized as one of the biological foundations of post-stroke late-onset depression and cognitive impairment (Mohamud Yusuf et al., 2024; Zhou et al., 2025). Although reperfusion is essential for restoring cerebral blood flow, it paradoxically induces secondary injury cascades, including excessive reactive oxygen species (ROS) production (Granger and Kvietys, 2015), persistent neuroinflammation driven by microglial activation, blood–brain barrier disruption, mitochondrial dysfunction, and energy metabolism imbalance (Hu et al., 2015; Wang et al., 2022). These pathological events synergistically exacerbate neuronal injury, impair synaptic integrity, and compromise the brain’s adaptive capacity to ischemic stress, ultimately contributing to long-term cognitive and affective disorders.

In recent years, microRNAs have emerged as critical regulators in ischemic brain injury and neurodegenerative processes. Accumulating evidence indicates that miRNAs participate not only in the acute phase of cerebral ischemia but also in chronic cerebral hypoperfusion and vascular cognitive impairment (Toyama et al., 2018; Zhang et al., 2020). For instance, miR-134-5p has been reported to contribute to cognitive deficits in a rat model of chronic cerebral ischemia by inducing cortical neuronal damage and synaptic protein loss (Liu et al., 2019). miR-26b contributes to microglial activation and consequent neurotoxicity under hypoxic/ischemic conditions by targeting the IL-6 pathway (Kang et al., 2018). Previous clinical studies have identified upregulated miR-140-5p in the blood of acute ischemic stroke patients, supporting its close association with the disease and its potential as a circulating biomarker (Sørensen et al., 2014). Notably, miR-140-5p has been reported to be closely associated with aging and cognitive decline (Toury et al., 2022). Our previous clinical and in vivo studies further revealed that miR-140-5p expression is significantly upregulated in patients with post-stroke late-onset depression (Liang et al., 2019), and that miR-140-5p overexpression aggravates cognitive impairment and inhibits neurogenesis in ischemic mouse models (Liang et al., 2022). These findings suggest that miR-140-5p may serve as an important molecular link between cerebral ischemia and long-term neurological dysfunction.

Consistent with our observations, prior studies in non-neuronal systems have shown that inhibition of miR-140-5p alleviates hypoxia-induced injury in H9c2 cardiomyocytes, whereas miR-140-5p aggravates cellular damage by activating stress-related MAPK pathways, including p38 MAPK and JNK, through regulation of MLK3 (Xing et al., 2018). miR-140-5p also promotes myocardial oxidative stress by targeting Nrf2 and Sirt2 (Zhao et al., 2017). Extending these findings to the central nervous system, our study provides direct evidence that miR-140-5p promotes JNK pathway activation in neurons under OGD/R conditions. Importantly, we identified IL1RAPL1 as a direct functional target of miR-140-5p. IL1RAPL1 is a synapse-associated protein involved in neuronal survival, synaptic development, and plasticity, and its suppression may sensitize neurons to inflammatory and apoptotic stimuli. The downregulation of IL1RAPL1 by miR-140-5p thus represents a critical molecular event linking post-ischemic inflammation, neuronal death, and synaptic dysfunction.

IL1RAPL1 is a synapse-associated protein belonging to the IL-1 receptor family, sharing approximately 52% homology with IL1RAP, and is highly expressed in brain regions critically involved in cognition and emotion, including the hippocampus, dentate gyrus, and entorhinal cortex (Gambino et al., 2007; Pavlowsky et al., 2010a). Functionally, IL1RAPL1 plays a pivotal role in synaptic development and plasticity by mediating excitatory synapse formation through trans-synaptic interactions with protein tyrosine phosphatase δ(PTPδ) (Takahashi and Craig, 2013; Yamagata et al., 2015). Notably, the synaptic role of IL1RAPL1 is not restricted to the hippocampus. In cultured cortical neurons, IL1RAPL1 has been shown to promote excitatory synapse formation and dendritic protrusion formation through trans-synaptic interaction with PTPδ, whereas IL1RAPL1 knockdown produces the opposite effects (Yoshida et al., 2011). IL1RAPL1 has also been shown to regulate the formation and stabilization of glutamatergic synapses in cortical neurons (Hayashi et al., 2013). Consistent with these in vitro findings, IL1RAPL1 knockout mice exhibit reduced cortical neuronal spine density, further supporting an important role for IL1RAPL1 in cortical synaptic organization. IL1RAPL1 knockout mice show decreased spine density, learning deficits, hyperactivity and reduced anxiety-like behaviors (Yasumura et al., 2014). Previous studies have further demonstrated that IL1RAPL1 regulates JNK activity and JNK-dependent phosphorylation of PSD-95, thereby modulating the synaptic localization of PSD-95 (Pavlowsky et al., 2010a). Moreover, IL1RAPL1 has been reported to mediate IL-1-induced JNK activation in neurons (Pavlowsky et al., 2010b). These findings provide a mechanistic basis for the functional link between IL1RAPL1 and JNK signaling. Consequently, loss of IL1RAPL1 function results in structural and functional excitatory synaptic deficits in the hippocampus, including decreased spine density and impaired long-term potentiation (Pavlowsky et al., 2010a). Based on its established roles in synaptic development and plasticity, together with the present findings showing increased IL1RAPL1 expression and improved neuronal viability following miR-140-5p inhibition, IL1RAPL1 may contribute to the maintenance of synaptic integrity and neuronal survival under OGD/R conditions. Consistent with the present findings, our previous study demonstrated that AAV-mediated overexpression of miR-140-5p significantly reduced the protein levels of IL1RAP, IL1RAPL1, VEGF, and MEGF10 in the hippocampus of ischemic mice, accompanied by impaired neurogenesis and decreased capillary density (Liang et al., 2019). Together with the present findings, these observations provide in vivo support for the involvement of IL1RAPL1 in miR-140-5p-associated neuronal and synaptic alterations following ischemic injury. In the present study, the identification of IL1RAPL1 as a direct target of miR-140-5p, together with the increased IL1RAPL1 expression and improved neuronal viability following miR-140-5p inhibition, supports the involvement of IL1RAPL1 in OGD/R-induced neuronal injury. Nevertheless, the precise functional contribution of IL1RAPL1 to the protective effects associated with miR-140-5p inhibition and its relationship with JNK signaling require further investigation.

Synaptic plasticity is a fundamental mechanism underlying the brain’s adaptive response to ischemic injury. However, this adaptive capacity is often impaired following stroke (Murphy and Corbett, 2009; Singh et al., 2025). Substantial evidence indicates that hippocampal neurogenesis, axonal branching, dendritic remodeling, and synapse formation are closely associated with chronic stress, affective disorders, and antidepressant efficacy, particularly in post-stroke depression (Verma et al., 2017; Yang et al., 2021). Synaptophysin is a 38 kDa transmembrane glycoprotein predominantly localized to presynaptic vesicle membranes and is widely recognized as a canonical marker of synaptic vesicles. It plays a critical role in maintaining synaptic structural stability and is involved in synaptic vesicle biogenesis, trafficking, and neurotransmitter release. Structurally, synaptophysin contains four transmembrane domains and forms hexameric complexes that interact with other synaptic vesicle–associated proteins. Through coordinated interactions with synaptic proteins such as syntaxin, synaptophysin regulates the fusion of synaptic vesicles with the presynaptic membrane, thereby facilitating neurotransmitter release (Masliah and Terry, 1993; Wiedenmann and Franke, 1985). Importantly, synaptophysin expression is closely associated with synaptic plasticity and is commonly used as an indicator of synaptic density and functional integrity (Gordon and Cousin, 2014). During neurodevelopment, synaptophysin expression increases in parallel with synapse formation and maturation, whereas its dysregulation has been linked to synaptic dysfunction in several neurological disorders, including Alzheimer’s disease and Parkinson’s disease (Soni and Shah, 2025). Reduced synaptophysin expression has also been reported in specific cortical regions of patients with schizophrenia (Eastwood et al., 2000). In addition, age-related loss of synaptophysin in the neocortex and hippocampus has been observed in mice and is accompanied by memory impairment (Zhong et al., 2008). Consistently, aging is associated with decreased levels of both the postsynaptic marker PSD-95 and the presynaptic marker synaptophysin, leading to synaptic integrity loss and cognitive decline (Bobkova et al., 2015; Chiang et al., 2019). Dlg4 encodes postsynaptic density protein 95 (PSD-95), a major postsynaptic scaffolding protein that is essential for the clustering of glutamate receptors and the regulation of synaptic plasticity. The reduction in PSD-95 levels is recognized as a key pathological feature of brain aging and major neurodegenerative disorders, such as Alzheimer’s and Huntington’s diseases (Bustos et al., 2017). As the most abundant scaffold protein within excitatory postsynaptic densities, PSD-95 interacts with a wide range of neuronal functional molecules, including glutamate receptors, signaling proteins, adhesion molecules, cytoskeletal proteins, and other scaffolding proteins, thereby orchestrating synaptic signaling and structural organization. Acting as a key adaptor protein, PSD-95 functionally bridges glutamatergic, dopaminergic, and serotonergic neurotransmission, enabling coordinated regulation of these systems at the postsynaptic level. In the present study, treatment with the inhibitor under OGD/R conditions significantly increased the expression of PSD-95 and synaptophysin, suggesting a protective effect on synaptic structure and function. These findings indicate that miR-140-5p–mediated suppression of IL1RAPL1 and activation of JNK signaling may represent a critical molecular mechanism linking ischemic injury to synaptic dysfunction and depressive-like behaviors.

In addition, inflammatory cytokines play a central regulatory role in the initiation and progression of ischemic stroke and ischemia–reperfusion (I/R) injury (Kumari et al., 2024). Although reperfusion restores oxygen and nutrient supply to ischemic tissue, it simultaneously triggers a robust and sustained inflammatory response, thereby leading to secondary neuronal damage and dysfunction of neural networks. In this process, the dynamic balance between pro-inflammatory and anti-inflammatory cytokines is considered a critical determinant of the severity of I/R injury and subsequent neurological recovery (Lu and Wen, 2025). Among these cytokines, interleukin-6 (IL-6) has been identified as a key mediator within the circulating cytokine network and is capable of responding rapidly to acute cerebral ischemia. Elevated serum IL-6 levels have been shown to correlate closely with stroke severity, infarct volume, and clinical outcomes, suggesting its potential value as a prognostic biomarker for stroke (Hall et al., 2025; Li et al., 2022; Pawluk et al., 2025). In contrast, interleukin-10 (IL-10), an important anti-inflammatory cytokine, exerts protective effects by limiting excessive inflammatory responses, attenuating secondary neuronal injury, and promoting post-injury repair processes. Previous studies have demonstrated that IL-10 levels are not only closely associated with post-stroke inflammatory status but also serve as an independent predictor of post-stroke depression, with predictive value for stroke severity, activities of daily living, and functional outcomes (Chi et al., 2021). In the present study, we found that inhibition of miR-140-5p markedly attenuated the inflammatory response in neurons subjected to OGD/R, as evidenced by reduced levels of the pro-inflammatory cytokine IL-6 and a relative increase in the anti-inflammatory cytokine IL-10. These findings suggest that miR-140-5p may contribute to the amplification of neuroinflammation during ischemia–reperfusion injury by modulating the balance of inflammatory cytokines. Taken together with the previously reported aberrant expression of miR-140-5p in late-stage post-stroke depression and cognitive impairment, we speculate that miR-140-5p–mediated inflammatory dysregulation may represent an important molecular mechanism linking ischemic brain injury to post-stroke affective and cognitive dysfunction.

Although our previous in vivo study demonstrated that miR-140-5p overexpression aggravated cognitive impairment and impaired hippocampal neurogenesis following cerebral ischemia, the specific molecular mechanism investigated in the present study was characterized using in vitro neuronal models, which cannot fully recapitulate the complex cellular and physiological responses to cerebral ischemia–reperfusion injury in vivo. In addition, the present study primarily evaluated neuronal injury and synaptic alterations using cell viability assays and molecular markers, and further functional assessments of neuronal and synaptic activity are warranted to confirm the biological significance of these findings. Consequently, the involvement of IL1RAPL1 and JNK signaling in mediating the effects of miR-140-5p requires further in vivo validation. To this end, future studies using animal models are warranted to substantiate the physiological and pathophysiological relevance of this signaling pathway.

## 5 Conclusion

Our integrated in vitro approach identified interleukin-1 receptor accessory protein-like 1 (IL1RAPL1) as a direct downstream target of miR-140-5p. Under OGD/R conditions, miR-140-5p-mediated suppression of IL1RAPL1 was associated with JNK pathway activation, enhanced neuronal apoptosis and inflammatory responses, and impaired synaptic integrity. These findings suggest a potential miR-140-5p/IL1RAPL1/JNK regulatory mechanism involved in OGD/R-induced neuronal injury.

## Data Availability

The mass spectrometry proteomics data generated in this study are publicly available in iProX under accession number IPX0020360002 (https://www.iprox.cn/page/SSV024.html;url=17920868432549WKs, password: Y9Rq). All other raw data supporting the conclusions of this study are available from the corresponding author upon reasonable request.

## References

[ref1] Bobkova N. V. Evgen'ev M. Garbuz D. G. Kulikov A. M. Morozov A. Samokhin A. . (2015). Exogenous Hsp70 delays senescence and improves cognitive function in aging mice. Proc. Nat. Acad. Sci. U.S.A. 112, 16006–16011. doi: 10.1073/pnas.1516131112 PMC4702952 26668376

[ref2] Bustos F. J. Ampuero E. Jury N. Aguilar R. Falahi F. Toledo J. . (2017). Epigenetic editing of the Dlg4/PSD95 gene improves cognition in aged and Alzheimer's disease mice. Brain J. Neurol. 140, 3252–3268. doi: 10.1093/brain/awx272, 29155979. PMC5841035

[ref3] Chi C.-H. Huang Y.-Y. Ye S.-Z. Shao M.-M. Jiang M.-X. Yang M.-Y. . (2021). Interleukin-10 level is associated with post-stroke depression in acute ischaemic stroke patients. J. Affect. Disord. 293, 254–260. doi: 10.1016/j.jad.2021.06.037, 34217963.

[ref4] Chiang A. C. A. Huo X. Kavelaars A. Heijnen C. J. (2019). Chemotherapy accelerates age-related development of tauopathy and results in loss of synaptic integrity and cognitive impairment. Brain Behav. Immun. 79, 319–325. doi: 10.1016/j.bbi.2019.04.005, 30953771. PMC6591052

[ref5] Eastwood S. L. Cairns N. J. Harrison P. J. (2000). Synaptophysin gene expression in schizophrenia. Investigation of synaptic pathology in the cerebral cortex. Br. J. Psychiatry J. Ment. Sci. 176, 236–242. doi: 10.1192/bjp.176.3.236, 10755070.

[ref6] Fang M. Zhong L. Jin X. Cui R. Yang W. Gao S. . (2019). Effect of inflammation on the process of stroke rehabilitation and poststroke depression. Front. Psychiatry 10:184. doi: 10.3389/fpsyt.2019.00184, 31031649. PMC6470379

[ref7] Gambino F. Pavlowsky A. Béglé A. Dupont J.-L. Bahi N. Courjaret R. . (2007). IL1-receptor accessory protein-like 1 (IL1RAPL1), a protein involved in cognitive functions, regulates N-type Ca2+-channel and neurite elongation. Proc. Nat. Acad. Sci. U.S.A. 104, 9063–9068. doi: 10.1073/pnas.0701133104, 17502602. PMC1885628

[ref8] Gendosz de Carrillo D. Kocikowska O. Krzan A. Student S. Rak M. Nowak-Andraka M. . (2025). Reduced expression of selected exosomal microRNAs is associated with poor outcomes in patients with acute stroke receiving reperfusion therapy—preliminary study. Int. J. Mol. Sci. 26:9533. doi: 10.3390/ijms26199533, 41096799. PMC12524926

[ref9] Gordon S. L. Cousin M. A. (2014). The Sybtraps: control of synaptobrevin traffic by synaptophysin, α-synuclein and AP-180. Traffic (Cph. Den.) 15, 245–254. doi: 10.1111/tra.12140, 24279465. PMC3992847

[ref10] Granger D. N. Kvietys P. R. (2015). Reperfusion injury and reactive oxygen species: the evolution of a concept. Redox Biol. 6, 524–551. doi: 10.1016/j.redox.2015.08.020, 26484802. PMC4625011

[ref11] Hall C. Nguyen D. T. Mendoza K. Tan C. Chauhan A. (2025). Inhibition of IL-6 trans-signaling promotes post-stroke functional recovery in a sex and dose-dependent manner. J. Neuroinflammation 22:52. doi: 10.1186/s12974-025-03365-y, 40011978. PMC11866694

[ref12] Hansen L. M. B. Dam V. S. Guldbrandsen H. Ø. Staehr C. Pedersen T. M. Kalucka J. M. . (2025). Spatial transcriptomics and proteomics profiling after ischemic stroke reperfusion: insights into vascular alterations. Stroke 56, 1036–1047. doi: 10.1161/STROKEAHA.124.048085, 40052263.

[ref13] Hayashi T. Yoshida T. Ra M. Taguchi R. Mishina M. (2013). IL1RAPL1 associated with mental retardation and autism regulates the formation and stabilization of glutamatergic synapses of cortical neurons through RhoA signaling pathway. PLoS One 8:e66254. doi: 10.1371/journal.pone.0066254 PMC3681934 23785489

[ref14] Hu Y. Deng H. Xu S. Zhang J. (2015). MicroRNAs regulate mitochondrial function in cerebral ischemia-reperfusion injury. Int. J. Mol. Sci. 16, 24895–24917. doi: 10.3390/ijms161024895, 26492239. PMC4632781

[ref15] Jurcau A. Ardelean A. I. (2022). Oxidative stress in ischemia/reperfusion injuries following acute ischemic stroke. Biomedicines 10:574. doi: 10.3390/biomedicines10030574, 35327376. PMC8945353

[ref16] Kang Y.-C. Zhang L. Su Y. Li Y. Ren W.-L. Wei W.-S. (2018). MicroRNA-26b regulates the microglial inflammatory response in hypoxia/ischemia and affects the development of vascular cognitive impairment. Front. Cell. Neurosci. 12:154. doi: 10.3389/fncel.2018.00154, 29937716. PMC6002499

[ref17] Kumari S. Dhapola R. Sharma P. Nagar P. Medhi B. HariKrishnaReddy D. (2024). The impact of cytokines in neuroinflammation-mediated stroke. Cytokine Growth Factor Rev. 78, 105–119. doi: 10.1016/j.cytogfr.2024.06.002, 39004599.

[ref18] Li J. Lin J. Pan Y. Wang M. Meng X. Li H. . (2022). Interleukin-6 and YKL-40 predicted recurrent stroke after ischemic stroke or TIA: analysis of 6 inflammation biomarkers in a prospective cohort study. J. Neuroinflammation 19:131. doi: 10.1186/s12974-022-02467-1, 35761288. PMC9235241

[ref19] Liang H.-B. He J.-R. Tu X.-Q. Ding K.-Q. Yang G.-Y. Zhang Y. . (2019). MicroRNA-140-5p: a novel circulating biomarker for early warning of late-onset post-stroke depression. J. Psychiatr. Res. 115, 129–141. doi: 10.1016/j.jpsychires.2019.05.018, 31129437.

[ref20] Liang H.-B. Lai Z.-H. Tu X.-Q. Ding K.-Q. He J.-R. Yang G.-Y. . (2022). MicroRNA-140-5p exacerbates vascular cognitive impairment by inhibiting neurogenesis in the adult mouse hippocampus after global cerebral ischemia. Brain Res. Bull. 183, 73–83. doi: 10.1016/j.brainresbull.2022.03.001, 35248684.

[ref21] Liu X. Zhang R. Wu Z. Si W. Ren Z. Zhang S. . (2019). miR‑134‑5p/Foxp2/Syn1 is involved in cognitive impairment in an early vascular dementia rat model. Int. J. Mol. Med. 44, 1729–1740. doi: 10.3892/ijmm.2019.4331, 31545395. PMC6777691

[ref22] Lochhead J. J. Ronaldson P. T. Davis T. P. (2024). The role of oxidative stress in blood-brain barrier disruption during ischemic stroke: antioxidants in clinical trials. Biochem. Pharmacol. 228:116186. doi: 10.1016/j.bcp.2024.116186, 38561092. PMC11410550

[ref23] Lu W. Wen J. (2025). Metabolic reprogramming and astrocytes polarization following ischemic stroke. Free Radic. Biol. Med. 228, 197–206. doi: 10.1016/j.freeradbiomed.2025.01.002 39756488

[ref24] Masliah E. Terry R. (1993). The role of synaptic proteins in the pathogenesis of disorders of the central nervous system. Brain Pathol. (Zurich Switz.) 3, 77–85. doi: 10.1111/j.1750-3639.1993.tb00728.x, 8269086.

[ref25] Mohamud Yusuf A. Borbor M. Hussner T. Weghs C. Kaltwasser B. Pillath-Eilers M. . (2024). Acid sphingomyelinase inhibition induces cerebral angiogenesis post-ischemia/reperfusion in an oxidative stress-dependent way and promotes endothelial survival by regulating mitochondrial metabolism. Cell Death Dis. 15:650. doi: 10.1038/s41419-024-06935-9, 39231943. PMC11374893

[ref26] Murphy T. H. Corbett D. (2009). Plasticity during stroke recovery: from synapse to behaviour. Nature Rev. Neurosci. 10, 861–872. doi: 10.1038/nrn2735 19888284

[ref27] Pavlowsky A. Gianfelice A. Pallotto M. Zanchi A. Vara H. Khelfaoui M. . (2010a). A postsynaptic signaling pathway that may account for the cognitive defect due to IL1RAPL1 mutation. Curr. Biol. 20, 103–115. doi: 10.1016/j.cub.2009.12.030, 20096586.

[ref28] Pavlowsky A. Zanchi A. Pallotto M. Giustetto M. Chelly J. Sala C. . (2010b). Neuronal JNK pathway activation by IL-1 is mediated through IL1RAPL1, a protein required for development of cognitive functions. Commun. Integr. Biol. 3, 245–247. doi: 10.4161/cib.3.3.11414, 20714405. PMC2918768

[ref29] Pawluk H. Woźniak A. Tafelska-Kaczmarek A. Kosinska A. Pawluk M. Sergot K. . (2025). The role of IL-6 in ischemic stroke. Biomolecules 15:470. doi: 10.3390/biom15040470, 40305179. PMC12024898

[ref30] Singh A. Dhalayat K. Dhobale S. V. Ghosh B. Datta A. Borah A. . (2025). Unravelling the brain resilience following stroke: from injury to rewiring of the brain through pathway activation, drug targets, and therapeutic interventions. Ageing Res. Rev. 109:102780. doi: 10.1016/j.arr.2025.102780, 40409413.

[ref31] Soni R. Shah J. (2025). Unveiling the significance of synaptic proteins in Parkinson's pathogenesis: a review. Int. J. Biol. Macromol. 304:140789. doi: 10.1016/j.ijbiomac.2025.140789, 39924013.

[ref32] Sørensen S. S. Nygaard A.-B. Nielsen M.-Y. Jensen K. Christensen T. (2014). miRNA expression profiles in cerebrospinal fluid and blood of patients with acute ischemic stroke. Transl. Stroke Res. 5, 711–718. doi: 10.1007/s12975-014-0364-8, 25127724.

[ref33] Sun S. Li Z. Xiao Q. Tan S. Hu B. Jin H. (2023). An updated review on prediction and preventive treatment of post-stroke depression. Expert Rev. Neurother. 23, 721–739. doi: 10.1080/14737175.2023.2234081, 37427452.

[ref34] Takahashi H. Craig A. M. (2013). Protein tyrosine phosphatases PTPδ, PTPσ, and LAR: presynaptic hubs for synapse organization. Trends Neurosci. 36, 522–534. doi: 10.1016/j.tins.2013.06.002, 23835198. PMC3789601

[ref35] Toury L. Frankel D. Airault C. Magdinier F. Roll P. Kaspi E. (2022). miR-140-5p and miR-140-3p: key actors in aging-related diseases? Int. J. Mol. Sci. 23:11439. doi: 10.3390/ijms231911439, 36232738. PMC9570089

[ref36] Toyama K. Spin J. M. Deng A. C. Huang T.-T. Wei K. Wagenhäuser M. U. . (2018). MicroRNA-mediated therapy modulating blood-brain barrier disruption improves vascular cognitive impairment. Arterioscler. Thromb. Vasc. Biol. 38, 1392–1406. doi: 10.1161/ATVBAHA.118.310822, 29650692.

[ref37] Tuo Q.-Z. Zhang S.-T. Lei P. (2021). Mechanisms of neuronal cell death in ischemic stroke and their therapeutic implications. Med. Res. Rev. 42, 259–305. doi: 10.1002/med.21817 33957000

[ref38] Verma R. Cronin C. G. Hudobenko J. Venna V. R. McCullough L. D. Liang B. T. (2017). Deletion of the P2X4 receptor is neuroprotective acutely, but induces a depressive phenotype during recovery from ischemic stroke. Brain Behav. Immun. 66, 302–312. doi: 10.1016/j.bbi.2017.07.155, 28751018. PMC5650951

[ref39] Wang Y. Leak R. K. Cao G. (2022). Microglia-mediated neuroinflammation and neuroplasticity after stroke. Front. Cell. Neurosci. 16:980722. doi: 10.3389/fncel.2022.980722, 36052339. PMC9426757

[ref40] Wiedenmann B. Franke W. W. (1985). Identification and localization of synaptophysin, an integral membrane glycoprotein of Mr 38,000 characteristic of presynaptic vesicles. Cell 41, 1017–1028. doi: 10.1016/S0092-8674(85)80082-9, 3924408.

[ref41] Xing B. Li Q.-J. Li H. Chen S.-S. Cui Z.-Y. Ma J. . (2018). miR-140-5p aggravates hypoxia-induced cell injury via regulating MLK3 in H9c2 cells. Biomed. Pharmacother. 103, 1652–1657. doi: 10.1016/j.biopha.2018.04.062, 29864954.

[ref42] Yamagata A. Yoshida T. Sato Y. Goto-Ito S. Uemura T. Maeda A. . (2015). Mechanisms of splicing-dependent trans-synaptic adhesion by PTPδ-IL1RAPL1/IL-1RAcP for synaptic differentiation. Nature Commun. 6:6926. doi: 10.1038/ncomms7926, 25908590. PMC4423211

[ref43] Yang B. Wang L. Nie Y. Wei W. Xiong W. (2021). proBDNF expression induces apoptosis and inhibits synaptic regeneration by regulating the RhoA-JNK pathway in an in vitro post-stroke depression model. Transl. Psychiatry 11:578. doi: 10.1038/s41398-021-01667-2, 34759285. PMC8580986

[ref44] Yasumura M. Yoshida T. Yamazaki M. Abe M. Natsume R. Kanno K. . (2014). IL1RAPL1 knockout mice show spine density decrease, learning deficiency, hyperactivity and reduced anxiety-like behaviours. Sci. Rep. 4:6613. doi: 10.1038/srep06613, 25312502. PMC4196104

[ref45] Yoshida T. Yasumura M. Uemura T. Lee S. J. Ra M. Taguchi R. . (2011). IL-1 receptor accessory protein-like 1 associated with mental retardation and autism mediates synapse formation by trans-synaptic interaction with protein tyrosine phosphatase delta. J. Neurosci. 31, 13485–13499. doi: 10.1523/JNEUROSCI.2136-11.2011 PMC6623287 21940441

[ref46] Zhang J. Sun P. Zhou C. Zhang X. Ma F. Xu Y. . (2020). Regulatory microRNAs and vascular cognitive impairment and dementia. CNS Neurosci. Ther. 26, 1207–1218. doi: 10.1111/cns.13472, 33459504. PMC7702235

[ref47] Zhao L. Hu M. Yin X. Yang H. Zhu L. Sun P. . (2025). Multifunctional miRNA delivery systems provide synergistic neuroprotection against cerebral ischemia reperfusion injury. Mater. Horizons 12, 9679–9696. doi: 10.1039/d5mh00875a, 40813285.

[ref48] Zhao L. Qi Y. Xu L. Tao X. Han X. Yin L. . (2017). MicroRNA-140-5p aggravates doxorubicin-induced cardiotoxicity by promoting myocardial oxidative stress via targeting Nrf2 and Sirt2. Redox Biol. 15, 284–296. doi: 10.1016/j.redox.2017.12.013, 29304479. PMC5975069

[ref49] Zhong N. Scearce-Levie K. Ramaswamy G. Weisgraber K. H. (2008). Apolipoprotein E4 domain interaction: synaptic and cognitive deficits in mice. Alzheimers Demen. 4, 179–192. doi: 10.1016/j.jalz.2008.01.006, 18631967. PMC2647370

[ref50] Zhou M. Tao X. Lin K. Leng C. Yang Y. Gui Y. . (2025). Downregulation of the HCN1 channel alleviates anxiety- and depression-like behaviors in mice with cerebral ischemia–reperfusion injury by suppressing the NLRP3 inflammasome. J. Am. Heart Assoc. 14:e038263. doi: 10.1161/jaha.124.038263, 40207529. PMC12132894

